# The Additional Use of Spirometry in Evaluating Chronic Cough in Children. “May the Force Be With You”

**DOI:** 10.7759/cureus.78441

**Published:** 2025-02-03

**Authors:** Vasilis Grammeniatis, Kostas Priftis, Konstantinos Douros

**Affiliations:** 1 Paediatrics, General Hospital of Ioannina G. Hatzikosta, Ioannina, GRC; 2 Third Department of Pediatrics, National and Kapodistrian University of Athens School of Medicine, Athens, GRC

**Keywords:** children, chronic cough, diagnosis, lung secretions, spirometry, tracheomalacia

## Abstract

Spirometry is the most common and straightforward examination following the mandatory initial steps of personal history and physical examination when assessing chronic and/or recurrent lung symptoms in children, especially cough or specific conditions that can impact lung function.

When dealing with a chronic cough (lasting more than four weeks), it is not uncommon to find that no specific clues regarding the cause of the cough can be deduced from the patient's history alone. Moreover, clinical examinations can be quite normal without any abnormal lung sounds. In the next step (spirometry), surprisingly, as the child forcefully expels the air from the lungs, you can hear secretions moving along the bronchi as the rapidly moving air drifts excessive sputum to the upper airways. Less commonly, forceful exhalation during spirometry may uncover a brassy or honking sound, indicating potential collapsibility of the tracheal walls.

Secretions in the bronchi can imply specific conditions, such as protracted bacterial bronchitis or suppurative bronchitis. Additionally, forced exhalation may uncover sputum in other chronic lung conditions, such as cystic fibrosis or primary cilia dyskinesia. The presence of tracheomalacia is an important parameter, as it can precipitate several respiratory symptoms, ranging from prolonged cough in acute bronchitis to recurrent and/or chronic wet cough.

In conclusion, forceful exhalation during spirometry has the potential to uncover secretions in the bronchi or even tracheomalacia that might otherwise go unnoticed. Before watching the flow-volume loop or interpreting the results of the spirometry parameters, we should first "hear" the spirometry.

## Editorial

Spirometry is the most common and straightforward examination following history and physical examination when assessing children's chronic or recurrent lung symptoms. It is particularly useful for evaluating cough or specific conditions that can impact lung function. Spirometry can generally be efficiently performed in most children aged six years and older and occasionally in younger children as well. It can measure how much air and how quickly it can be exhaled in a single blow from both lungs, starting from full inhalation.

Airflow during forced expiration (or inspiration) depends on the lung's elastic recoil, the airways' resistance, and the respiratory muscles' strength. Expiratory flow can be reduced by airway narrowing, such as from bronchoconstriction, while abnormal inspiratory flow may indicate upper airway dysfunction [1].

Spirometry can measure several parameters of lung function, including forced expiratory volume in 1s (FEV1), forced vital capacity (FVC), and the ratio of FEV1/FVC. Assessing such parameters and evaluating reversibility after a bronchodilator allows for estimating patterns of obstructive, restrictive, or mixed lung diseases [2].

Spirometry is the cornerstone tool for diagnosing and monitoring asthma, one of the most common airway diseases, affecting as much as 10% of the pediatric population [3]. However, asthma is primarily a wheezing rather than a coughing disorder. When addressing chronic cough as the main symptom, obtaining reliable information from the history is often challenging, as parents may struggle to accurately describe their child's symptoms. Cough characteristics, such as whether it is dry or wet, brassy or barking, and the timing (morning vs. night) along with the presence of wheezing or noisy breathing, may not be accurately assessed by parents, leading to incomplete or unclear descriptions. Moreover, clinical examination can be quite normal without abnormal lung sounds.

In the next step (spirometry), surprisingly, as the child forcefully expels the air from the lungs, you can hear secretions moving along the bronchi as the rapidly moving air drifts excessive sputum to the upper airways. When addressing the matter of this 'new' sound, the answer is, "Ah, I see what you're asking now. My child has had these secretions for a while, and sometimes it can expectorate colored sputum". Secretions in the bronchi can imply specific conditions, such as protracted bacterial bronchitis or suppurative bronchitis. Additionally, forced exhalation may uncover sputum in other chronic lung conditions, such as cystic fibrosis or primary cilia dyskinesia. This can lead to a more accurate assessment of the effectiveness of prior treatments for these diseases.

Less commonly, forceful exhalation during spirometry may uncover a brassy or honking sound, indicating potential collapsibility of the tracheal walls. This information may not be evident from the patient's previous medical history alone. The presence of tracheomalacia is an important parameter, as it can precipitate several respiratory symptoms, ranging from prolonged cough in acute bronchitis to recurrent and/or chronic wet cough [4]. Tracheomalacia can be challenging to diagnose based on spirometry results, as only about 25% of cases show a spirometry pattern suggestive of the disorder [5].

Altogether, excessive secretions and/or tracheomalacia can be identified during forceful exhalation in spirometry, offering a valuable indicator in the algorithm for diagnosing chronic cough disease. This simple procedure can be conducted in any respiratory office, enhancing diagnostic capabilities. Its importance is underscored by the frequent absence of abnormal findings in clinical examination. Additionally, there can be instances of miscommunication between physicians and caregivers, especially when caregivers use non-specific phrases in order to better describe the cough. These descriptions might not accurately convey the specific characteristics of the cough, such as whether it is wet, brassy, or accompanied by wheezing.

It is essential to consider that the child's cooperation is crucial during spirometry. The detection of excessive sputum or tracheomalacia relies on the velocity of the moving air during a forceful exhalation, which may not be achieved in some children, especially those at borderline ages, who may struggle to exert maximum effort.

In conclusion, forceful exhalation during spirometry has the potential to uncover existing secretions in the bronchi or even tracheomalacia, which might otherwise go unnoticed. This contributes to more effective management of chronic cough in children by assisting in diagnosing and monitoring underlying diseases. It is crucial for the physician to be present in the same room with the patient during spirometry since the technician may not be able to obtain and share such information. Before watching the flow-volume loop or interpreting the results of the spirometry parameters, we should first "hear" at the spirometry.
